# Critical Care Nurses' Perceptions and Experiences of the Organ Donation Process: A Systematic Review

**DOI:** 10.1002/nop2.70420

**Published:** 2026-01-05

**Authors:** Natcha Thilly Roth, Eva Åkerman

**Affiliations:** ^1^ Department of Thoracic Surgery Skåne University Hospital Lund Sweden; ^2^ Department of Health Sciences Lunds University Lund Sweden

**Keywords:** critical care nursing, experiences, organ donation, organ transplantation

## Abstract

**Aim:**

To describe critical care nurses' perceptions and experiences of the organ donation process.

**Design and Methods:**

A systematic review with a literature search in CINAHL, PubMed, and PsycINFO was conducted between 2009–2024 and reported by the PRISMA guidelines. 14 qualitative studies were assessed for relevance and methodological quality. Data were extracted by two of the researchers. A quality appraisal was performed according to a review template, first individually and then discussed in the research group. The analysis followed an interpretative synthesis, in three steps: coding, developing descriptive themes and generating analytical themes.

**Results:**

Four themes were identified: lack of knowledge, work environment, meeting with relatives and nurse's challenges. The nurses described that caring for a potential organ donor was complex. The nurses' general lack of knowledge about organ donation led to stress and feelings of uncertainty, which in turn created limitations in the donation process. Nurses identified several challenges with the organ donation process.

**Conclusion:**

The staff found the organ donation process challenging on various levels. There was a significant need for crisis processing for all professionals involved in the donation process.

**Implications:**

Addressing the knowledge gap through clear guidelines and internal training to enhance the organ donation process. Providing time for debriefing can help manage strain on professionals.

**Impact:**

This study highlights the critical need for improved education and emotional support for critical care nurses. Implementing these changes can lead to more efficient and compassionate organ donation processes.

## 1 Introduction

Organ transplantation has long been a life‐saving treatment for patients with end‐stage organ failure (Lewis et al. 2021). A problem with organ transplantation is that the supply of organs and tissues is lower than the demand. This imbalance between the supply and demand has led to a persisting high mortality rate among patients on the waiting list for an organ (Lewis et al. 2021).

When a patient's life cannot be saved, and life‐sustaining therapy is no longer effective, the attending physician can decide to withdraw the life‐sustaining therapy given for the patient's benefit (Smith et al. 2019). The continued intensive care then has a new purpose in preserving the function of organs for a potential donation, referred to as organ preservation treatment (Smith et al. 2019).

There are two kinds of donation processes, Donation after Brain Death (DBD) and Donation after Circulatory Death (DCD) (Domínguez‐Gil et al. 2011). DBD applies when the cause of death is a total cerebral infarction caused by a primary brain damage, and to keep the organs viable for donation it is necessary that the patient is kept on life support and that the procurement surgery is often performed within 24–48 h of the death, but it could also take up to a few days depending on the organs (Siddique and Mallat 2023; Eerola et al. 2022). DCD applies when death occurs as a result of circulatory arrest. The heart stops beating, and oxygenated blood no longer reaches the brain, resulting in total cerebral infarction (Thuong et al. 2016). The DCD process includes a decision of withdrawal of life‐sustaining treatment. Then after a permanent cessation of circulation (Gries et al. 2013), there will be a no‐touch period to ensure that autoresuscitation will not occur, after which procurement surgery initiates (Lomero et al. 2020). The critical care nurse (CCN) has a significant role in the donation process to care for potential donors, promoting organ donation, and providing support to relatives during the care of the donor (Jawoniyi and Gormley 2015).

**FIGURE 1:**
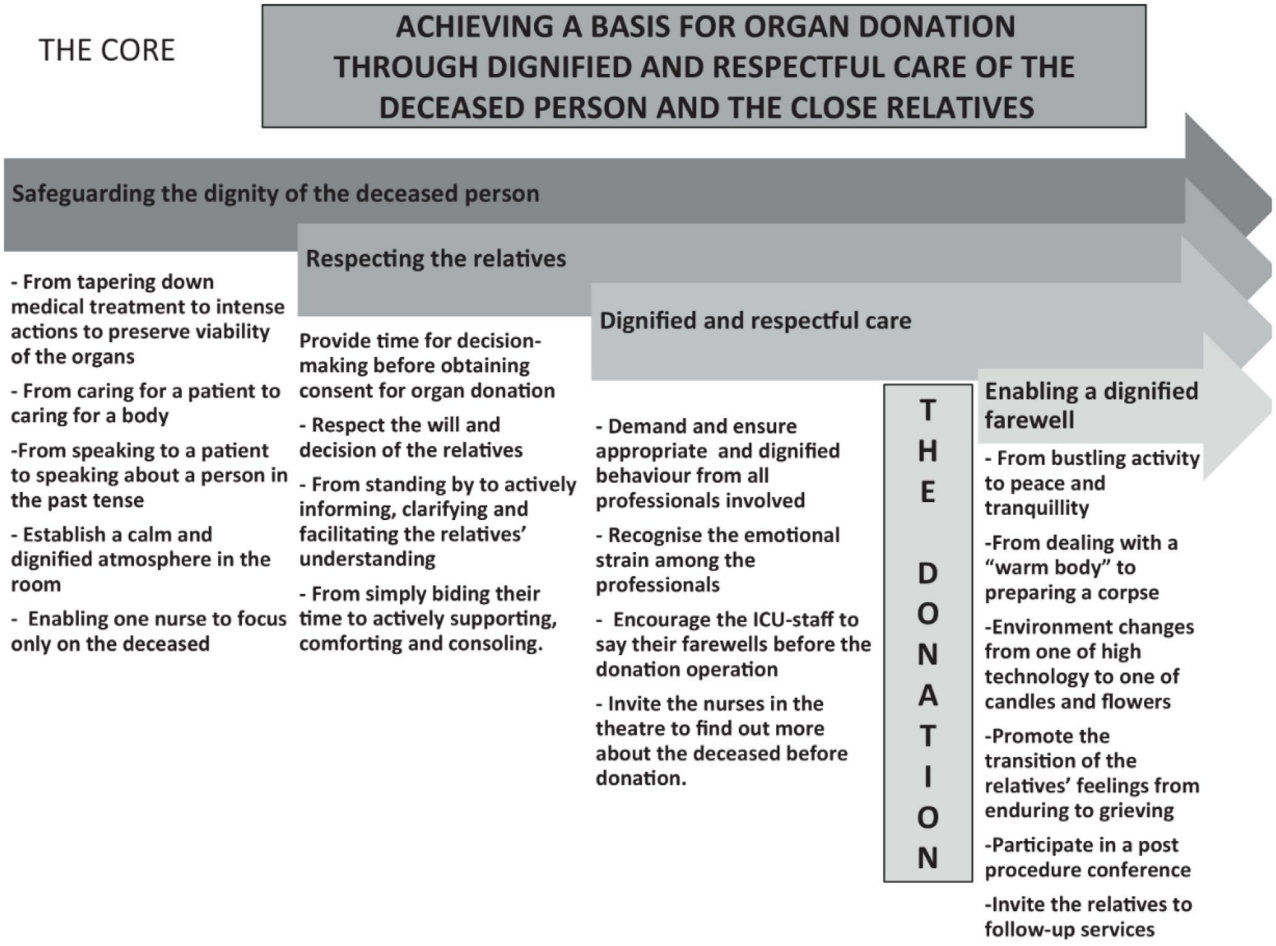
The process of organ donor care (Forsberg et al. 2014) is published with permission from Anna Forsberg.

Forsberg et al. (2014) conducted a study on CCNs' experiences and perceptions of caring for deceased organ donors. Based on the study's findings, they developed a model to describe the core of post‐mortem care in relation to organ donation (Figure 1). The model consists of four phases, each representing the core of the nursing process for a deceased organ donor, starting when the patient is declared dead and identified as a potential organ donor until the final farewell with the relatives after death. The model suggests that organ donation can be facilitated through respectful and dignified care of the deceased and their families (Forsberg et al. 2014).

Ethical dilemmas associated with organ donation can be a significant problem for the entire team (Araújo and Massarollo 2014; Jawoniyi et al. 2018; Sorensen et al. 2021). The donation can be emotionally challenging for CCN, as it places high demands on their professionalism and competence (Forsberg et al. 2014). Caring for an organ donor and their relatives is complex, and it requires good cooperation in the team to be able to meet them and their needs (Hunt and Murphy 2020). The knowledge among health care professionals about the donation process has to be high (Senna et al. 2020); then insufficient knowledge has been reported to jeopardise patient safety and may prevent CCN from carrying out their tasks in an adequate and secure manner (Jawoniyi and Gormley 2015; Senna et al. 2020). Then there is a discrepancy between patients in need of organ transplants and organ donors; the organisation and routines around the donation process need to be of high quality (Jawoniyi and Gormley 2015). Further research is required to understand what enhances the organ donor process and how to support the CCN. By conducting this systematic review and acquiring more knowledge from the CCNs' experience of organ donation, CCNs can be helped to prepare for the challenges that may emerge during the donation process and contribute to the ongoing advancement of this field. Therefore, it is important to describe the CCNs' perception and experiences of the donation process.

### 1.1 Aim

The aim was to describe CCNs perceptions and experiences of the donation process in an intensive care unit.

## 2 Methods

### 2.1 Design

This qualitative systematic review was reported according to the Preferred Reporting Items for Systematic Reviews (PRISMA) guidelines (Page et al. 2021) and by Bettany‐Saltikov and McSherry (2016) description.

### 2.2 Sample and Data Collection

The systematically searched were conducted in three databases: Cumulative Index Nursing and Allied Health (CINAHL), PubMED, and PsycINFO. The PEO, population (P) *nurses in intensive care units*, exposure (E) *organ donation*, Outcome (O) *experiences* was used to identify that the components in the review question were covered. The components were paraphrased into searchable terms or MeSH terms and combined with Boolean operators. The search strategy was developed by the researcher (NTR) and an experienced librarian. The search was undertaken in February 2024 and generated a total of 379 studies (Table 1).

**TABLE 1:** Database search in CINAHL, PubMed and PsycINFO.

Database	Block 1	Block 2	Block 3	Block 4	Results
Cinahl	Nurse* OR MH‘Nurses+’	MH‘Organ Donation’ OR organ donation OR MH‘Organ Transplantation+’ OR organ transplantation OR DCD OR DBD	MH‘Affect’ OR affect* OR MH‘Perception+’ OR perception OR MH‘Life Experiences+’ OR experience*	MH‘Intensive Care Units+’ OR intensive care OR MH‘Critical Care+’ OR critical care OR MH‘Critical Care Nursing+’ OR critical care nursing OR ICU	173
Pubmed	‘Nurse’[Mesh] OR nurse*	‘Tissue and organ procurement’ [Mesh] OR ‘Tissue and organ harvesting’ [Mesh] OR ‘Organ transplantation’ [Mesh] OR organ transplantation OR organ donation OR DCD OR DBD	‘Perception’ [Mesh] OR perception OR ‘Affect’ [Mesh] OR affect* OR ‘Life Change Events’ [Mesh] OR experience*	‘Critical care’ [Mesh] OR critical care OR ‘critical care nursing’ [Mesh] OR ‘intensive care units’ [Mesh] OR ICU	150
Psycinfo	Nurse* OR nursing	Organdonation OR Organ transplantation OR DE‘Organ Transplantation’ OR DCD OR DBD	DE‘Perception’ OR perception OR DE‘Life experiences’ OR experience* OR affect*	DE‘Intensive Care’ OR intensive care OR critical care OR critical care nursing OR ICU	56

### 2.3 Eligibility Criteria

Studies with a qualitative design, published in peer‐reviewed journal 2009–2024, written in English were included. The studies had to be conducted in an intensive care environment, and the participants were nurses with experience in intensive care. Studies focusing on the paediatric population and living donors were excluded.

### 2.4 Searching for Evidence and Selecting Studies

**FIGURE 2:**
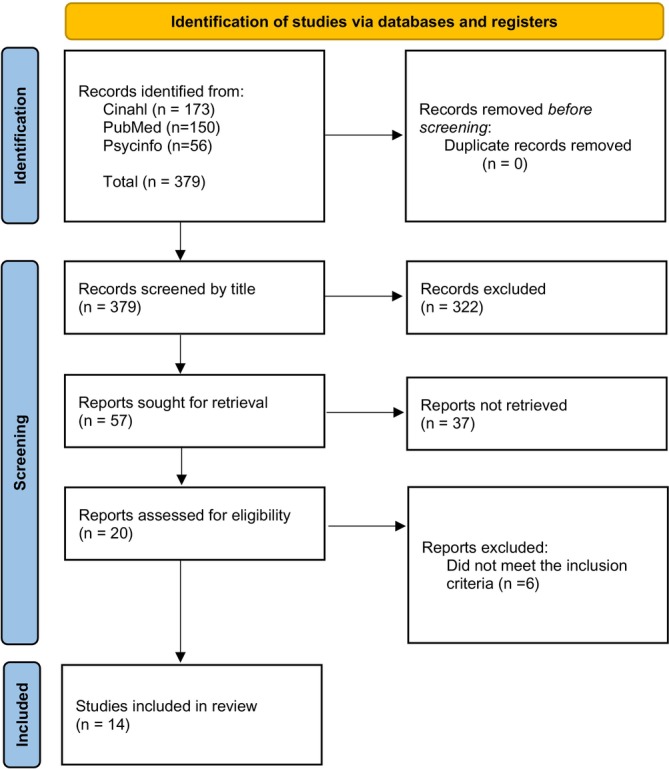
PRISMA 2020, flow chart for systematic reviews which included searches of databases and registers only.

The results from the database search were downloaded into a review template. The relevance assessment of each chosen article was first carried out individually and then discussed in the study group. The titles and abstracts were read and those articles not suitable for this study's aim, inclusion or exclusion criteria were excluded. A total of 20 articles were considered relevant and were read in full text and a full relevance assessment was made according to the Swedish Agency for Health Technology Assessment and Assessment of Social Services template for relevance assessment (SBU) including details of participants, exposure, and outcomes (SBU 2013). Six articles were ineligible based on this study's aim or inclusion criteria. A total of 14 articles were then advanced to the quality assessment (Figure 2).

### 2.5 Literature Quality Appraisal

Data extractions were conducted by two of the researchers and involved collecting information about the study titles, authors, publication years and countries, methodological characteristics as aim, design, participants, data collection and the studies' main conclusion. A quality appraisal was performed according to SBU's review template for qualitative studies (Table 2) (SBU 2022). The template includes five areas, with 13 methodological questions answered with yes, no, or unclear. The studies' quality was first individually evaluated and then discussed in the research group. The risk was assessed as low if more than one area was assessed as with no, medium if one area, and high if no area was assessed with no. All articles were deemed relevant, of satisfactory quality, and were therefore included (Table 2).

**TABLE 2:** Quality appraisal checklist according to SBU's review template for included studies.

Authors	Consistency between theoretical framework and methodology in the study	Significant deficiencies that could affect the reliability of the sampling process	Significant deficiencies that could affect reliability of the data collection process	Significant deficiencies that could affect reliability of the data analysis process	The researchers' background and competence are described
Flodén, Berg, and Forsberg (2011)	U	N	N	N	Y
Flodén and Forsberg (2009)	U	N	N	N	Y
Gripewall et al. (2022)	Y	N	N	N	Y
Moghaddam, Manzari, et al. (2018) and Moghaddam, Pouresmaili, and Manzari (2018)	Y	N	N	N	Y
Moghaddam et al. (2020)	U	N	N	N	Y
Orøy et al. (2015)	U	N	N	N	Y
Salehi et al. (2013)	U	N	N	N	Y
Simonsson et al. (2020)	Y	N	N	N	Y
Son and Kim (2024)	Y	N	N	N	Y
Souza et al. (2013a)	Y	N	N	N	Y
Souza et al. (2013b)	Y	N	N	N	Y
Starzomski et al. (2021)	U	N	N	N	Y
Victorino et al. (2019)	U	N	N	N	Y
Virgínio et al. (2014)	U	N	N	N	Y

*Note:* Y: Yes, N: No, U: Unclear.

### 2.6 Data Synthesis

The synthesis consisted of three steps: coding, developing descriptive themes and generating analytical themes. An inductive analysis was performed by repeatedly reading the findings, first individually and then together by the researchers. Sentence units relevant to the study's aim were identified and a data extraction with open coding was carried out. The sentence units were sorted into descriptive themes according to differences and similarities. The researchers went through all the sentence units and themes again and some minor adjustments were made, whereafter lists of subthemes and themes were developed. The final step of generating analytical themes was based on a discussion between the researchers where the descriptive themes were used in the process to go beyond the content of the original studies (Bettany‐Saltikov and McSherry 2016).

## 3 Results

### 3.1 Study Selection and Details

The search retrieved a total of 379 articles, of which 14 were reviewed in full and included (Figure 2). Eleven studies were of high quality according to the quality review; three articles were of medium quality (Table 3). The included studies were conducted in Sweden (4), Brazil (4), Iran (3), Canada (1), South Korea (1), and Norway (1) and published between 2009 and 2024. The sample size was between 7–115 and included a total of 305 participants. The majority of studies utilised semi‐structured interviews; one combined focus groups and individual interviews.

**TABLE 3:** Study characteristics.

Author(s)/year	Country/setting(s)	Participants	Data collection method	Key findings	Quality assessment
Flodén, Berg, and Forsberg (2011)	Sweden/ICU	15	Interview study	Ambiguity and different perceptions of brain death is affecting the quality of given care to the patient	High
Flodén and Forsberg (2009)	Sweden/ICU	9	Interview study	The chances of an potential donor becoming an actual donor depends on the ICU nurses' perception of organ donation	High
Gripewall et al. (2022)	Sweden/ICU	12	Interview study	Caring for an potential donor is a complex process involving communication, teamwork and organisation	High
Moghaddam, Manzari, et al. (2018)	Iran/ICU, transplant unit	21	Interview study	Nurses' care for potential donors promotes organ's viability for donation	High
Moghaddam et al. 2020	Iran/ICU	28	Interview study	Lacking education and managerial problems increased tension for nurses	High
Orøy et al. (2015)	Norway/ICU	32	Combined observation study and interviews	The prognostic process had a great influence on how healthcare professionals interacted with the family	High
Salehi et al. (2013)	Iran/ICU	8	Interview study	Caring for potential donors without suitable working conditions can be stressful and harmful to nurses' health	Medium
Simonsson et al. (2020)	Sweden/ICU	7	Interview study	Nurses regardless limited experienced or more experienced of caring for potential donors found their tasks to be highly demanding	High
Son and Kim (2024)	South Korea/ICU	10	Interview study	Nurses did not receive proper education on regulations related to caring of organ donors. They were instead self‐taught by senior nurses	High
Souza et al. (2013a)	Brazil/ICU	14	Interview study	Nurses' difficulties with identifying with the situation of potential donor is partly caused by doubt of brain death diagnosis	High
Souza et al. (2013b)	Brazil/ICU	14	Interview study	Organisational support is mandatory to provide support to nurses	High
Starzomski et al. (2021)	Canada/ICU	112	Interview study	To improve the organ donation process implementation of clinical guidelines was suggested. Collaborative practice models was encouraged to promote team work	High
Victorino et al. (2019)	Brazil/ICU	22	Interview study	Nurses' personal beliefs, culture and educational background influences their perceptions of organ donation	Medium
Virgínio et al. (2014)	Brazil/ICU	15	Interview study	Nurses finds it challenging to deal with organ donation process as it involves the process of dying	Medium

### 3.2 Thematic Findings

The data synthesis results in four themes: lack of knowledge, Communcation and Collaboration, meeting with relatives, and nurse's challenges.

#### 3.2.1 Lack of Knowledge

There was a considerable lack of knowledge among nurses, and this had negative effects on the caring related to the donation process (Flodén and Forsberg 2009; Flodén, Berg, and Forsberg 2011; Gripewall et al. 2022; Moghaddam et al. 2020; Salehi et al. 2013; Simonsson et al. 2020; Starzomski et al. 2021; Son and Kim 2024; Victorino et al. 2019).

#### 3.2.2 Not Having the Right Prerequisites

Nurses saw the care of a potential donor as a major challenge, and a reason for this was a lack of knowledge about the donation process and brain death diagnosis (Flodén and Forsberg 2009; Moghaddam et al. 2020). Many nurses had not received any training during their nursing school; instead, they had been taught afterwards when they started working (Son and Kim 2024; Starzomski et al. 2021). Some had been trained by their colleagues; others had engaged in self‐study (Salehi et al. 2013; Simonsson et al. 2020; Son and Kim 2024; Starzomski et al. 2021). Nurses with limited experience working with organ donation felt a responsibility to independently acquire knowledge for the care of a potential organ donor (Simonsson et al. 2020). The nurses felt that they needed internal training in the organ donation process, and preferably where they could be trained together with the other health care professions that were part of the team (Simonsson et al. 2020; Starzomski et al. 2021). An easily accessible and clear protocol with guidelines would have been beneficial to create security and increase safety (Simonsson et al. 2020).

#### 3.2.3 Doubts About the Brain Death Diagnosis

Not understanding fully what a brain death diagnosis meant created unease and doubt for nurses as they felt unsure if the patient was dead (Gripewall et al. 2022; Moghaddam et al. 2020; Salehi et al. 2013; Victorino et al. 2019). It was not uncommon for nurses to understand the physiology behind a brain death diagnosis, but it was difficult to understand it emotionally once they had a brain‐dead patient in front of them (Flodén, Berg, and Forsberg 2011). Doubts and mistrust about the diagnosis of brain death created conflicts between nurses and doctors as they disagreed on the level of care for the patient (Moghaddam et al. 2020). The nurses felt that it was wrong when the organs of these patients were donated. Nurses believed that they were causing the patient unnecessary suffering while waiting for their organs to be donated and that the patient was alive until the donation operation took place (Gripewall et al. 2022; Moghaddam et al. 2020; Souza et al. 2013b). There was a need to be present when doctors performed the diagnostic tests for brain death diagnosis as they felt more confident about the diagnosis if they could be involved in confirming the outcome (Flodén, Berg, and Forsberg 2011). For some nurses, an additional X‐ray examination to confirm that the patient was brain dead was required to convince them (Flodén, Berg, and Forsberg 2011).

#### 3.2.4 Negative Influences

The lack of knowledge created stress and a sense of uncertainty, which was experienced as frustrating as the care of this patient group was negatively affected by these feelings (Moghaddam et al. 2020). Planning the care was difficult as nurses did not always know how to prioritise, as there was a lot of pressure on nurses to keep the patient's organs in the best possible condition for the actual donation surgery (Moghaddam et al. 2020).

### 3.3 Communication and Collaboration

Having a successful collaboration with other professions in the team and receiving support from colleagues and managers were important factors for nurses' work environment (Flodén, Berg, and Forsberg 2011; Gripewall et al. 2022; Salehi et al. 2013; Simonsson et al. 2020; Starzomski et al. 2021).

#### 3.3.1 Importance of Teamwork

The work environment was expressed as an important factor and nurses felt that an open climate in the workplace and good communication with other professions in the team promoted the donation process (Gripewall et al. 2022; Starzomski et al. 2021). Caring for a potential donor required collaboration within the healthcare team, but some nurses felt that it was the role of the responsible physician to create a favourable environment for organ donation (Flodén, Berg, and Forsberg 2011; Starzomski et al. 2021). The physician's opinions and attitudes towards organ donation, as well as previous experience with organ donation, had a major impact on whether a donation would take place (Flodén, Berg, and Forsberg 2011; Starzomski et al. 2021). Conflicts could arise when a donation did not take place due to a lack of commitment from the physician; these situations could create frustration among nurses (Starzomski et al. 2021). Lack of communication in the team could cause conflicts. This could involve the physician acting in a particular way, such as talking to relatives or making decisions about the patient's care, without informing or involving the nurse and the rest of the team (Flodén, Berg, and Forsberg 2011).

#### 3.3.2 The Need for Debriefing

Nurses had experiences of both poor and good support from employers (Gripewall et al. 2022; Simonsson et al. 2020). There was a great need for debriefing and time for recovery after the emotional strain of working with the donation process (Simonsson et al. 2020). Crisis management was considered necessary even for those nurses where the experience of caring for a potential organ‐donor had not been negative (Flodén, Berg, and Forsberg 2011).

#### 3.3.3 Collegial Support

Nurses described that support from colleagues and managers was necessary to be able to cope with the stress (Gripewall et al. 2022; Salehi et al. 2013; Simonsson et al. 2020; Starzomski et al. 2021). Being able to reflect with colleagues was considered valuable as the nurses could vent their feelings and thus alleviate the emotional stress (Flodén, Berg, and Forsberg 2011; Simonsson et al. 2020; Starzomski et al. 2021). Working during daytime was beneficial; staffing was better and support from colleagues and managers was therefore more readily available (Starzomski et al. 2021). Good communication with the transplant coordinator also increased the nurses' sense of support and security, making the situation more manageable (Gripewall et al. 2022).

### 3.4 Meeting With Relatives

The care of a potential donor was broad and complex, and one of the major parts was the contact with the patient's relatives (Flodén and Forsberg 2009; Flodén, Berg, and Forsberg 2011; Gripewall et al. 2022; Moghaddam, Manzari, et al. 2018; Moghaddam et al. 2020; Orøy et al. 2015; Simonsson et al. 2020; Souza et al. 2013b). Nurses felt that the relationship with family members was unique and that it could have both positive and negative effects on the care (Flodén and Forsberg 2009; Flodén, Berg, and Forsberg 2011; Gripewall et al. 2022; Moghaddam, Manzari, et al. 2018; Moghaddam et al. 2020; Orøy et al. 2015; Simonsson et al. 2020; Souza et al. 2013b).

#### 3.4.1 Building a Relationship

Establishing a good relationship with the relatives was paramount for the key to success in the work with the donation process (Flodén and Forsberg 2009; Flodén, Berg, and Forsberg 2011). By being available, treating them with dignity, and being attentive, nurses could establish a strong connection with the patient's family (Flodén and Forsberg 2009; Flodén, Berg, and Forsberg 2011). Working closely with the patient's relatives could lead to a strong bond, and some nurses described the relationship with the relatives as emotional and touching (Gripewall et al. 2022). However, working with individuals in crisis was not always easy and could be demanding; thus, it was not always possible for nurses to provide the support the relatives needed (Souza et al. 2013b). Some nurses found it easier to perform certain activities related to nursing when the family was not present (Orøy et al. 2015). Continuity was highly valued by the relatives but could be burdensome for the nurses, although it enabled their ability to observe and confirm the support needed by the relatives (Simonsson et al. 2020).

#### 3.4.2 To Be Able to Communicate

Providing information about the brain death diagnosis and supporting the relatives was described as demanding and one of the most difficult tasks for nurses, especially for those with limited experience in caring for an organ‐donor (Simonsson et al. 2020). Nurses described a fear of displaying strong emotional reactions. They did not want to risk giving incorrect information to the relatives, as there have been situations where relatives blamed nurses for the patient's death (Moghaddam, Manzari, et al. 2018; Moghaddam et al. 2020). The nurses believed that it was a conversation that the attending physician should be responsible for (Moghaddam, Manzari, et al. 2018).

The complex situation could mean that a significant part of the nurses' focus was on the organ preservation treatment, and support for the relatives was sometimes forgotten. It was common to feel that they did not spend as much time with the relatives as they would have liked (Gripewall et al. 2022; Starzomski et al. 2021).

### 3.5 Nurse's Challenges

The work involved in the donation process could lead to both mental and physical stress for the nurse (Flodén and Forsberg 2009; Flodén, Berg, and Forsberg 2011; Gripewall et al. 2022; Moghaddam, Manzari, et al. 2018; Moghaddam et al. 2020; Orøy et al. 2015; Salehi et al. 2013; Simonsson et al. 2020; Souza et al. 2013a, 2013b). The nurses faced many challenges, and much of it could be linked to a lack of knowledge that did not provide the nurses with the right armament to handle the situation in a safe or secure way (Flodén and Forsberg 2009; Flodén, Berg, and Forsberg 2011; Gripewall et al. 2022; Moghaddam, Manzari, et al. 2018; Moghaddam et al. 2020; Orøy et al. 2015; Salehi et al. 2013; Simonsson et al. 2020; Souza et al. 2013a, 2013b). The care of a potential donor was perceived to be more burdensome and demanding compared to the care of a non‐donor patient (Flodén and Forsberg 2009; Flodén, Berg, and Forsberg 2011; Gripewall et al. 2022; Moghaddam, Manzari, et al. 2018; Moghaddam et al. 2020; Orøy et al. 2015; Salehi et al. 2013; Simonsson et al. 2020; Souza et al. 2013a, 2013b). Despite the negative aspects, there were positive aspects that nurses experienced during their work (Flodén and Forsberg 2009; Moghaddam et al. 2020; Simonsson et al. 2020; Souza et al. 2013b; Son and Kim 2024; Starzomski et al. 2021).

#### 3.5.1 The Emotional Reaction

Caring for a potential organ‐donor could evoke strong emotions, especially if the patient was young or if the patient's life situation resembled that of the nurses (Flodén and Forsberg 2009; Souza et al. 2013a). Not being adequately prepared to handle the emotional reactions of family members could also be experienced as stressful and frustrating (Salehi et al. 2013). Caring for a potential organ‐donor required more emotional involvement compared to other patients and could therefore be perceived as more emotionally demanding (Flodén, Berg, and Forsberg 2011; Simonsson et al. 2020). Nurses reported that due to the difficulties related to the situation, they experienced emotions such as grief, anxiety, sadness, despair, hopelessness, fear, helplessness, and powerlessness (Flodén and Forsberg 2009; Salehi et al. 2013; Souza et al. 2013a). Lack of support from colleagues could lead to feelings of abandonment and loneliness (Gripewall et al. 2022)

Despite some nurses having extensive experience in intensive care, caring for a potential organ‐donor was still perceived as unique, challenging, and uncomfortable (Moghaddam, Manzari, et al. 2018). Nurses could feel a sense of grief due to the circumstances because they sympathised with the patient's family members (Souza et al. 2013a, 2013b; Starzomski et al. 2021). Many nurses turned to their faith to cope with the challenges that could arise (Souza et al. 2013b). Others attempted to manage it by not becoming too emotionally involved and avoiding contact with family members (Souza et al. 2013b). The organ preservation treatment, which involved continued ventilation, oxygenation, and vasopressors, felt unethical to some and was described as an ethical dilemma (Orøy et al. 2015).

#### 3.5.2 A Physical Strain

Caring for a potential donor often meant an increased workload (Moghaddam et al. 2020). The challenges of caring for a potential organ‐donor were partly connected to the sensitive and vulnerable nature of the situation, but they were related to stress, lack of knowledge, and intense nursing care (Moghaddam, Manzari, et al. 2018; Moghaddam et al. 2020; Salehi et al. 2013). Many nurses could experience physical exhaustion. Outside of work they could have problems such as sleep difficulties and nightmares (Moghaddam et al. 2020; Souza et al. 2013a). This could negatively impact their job performance, leading to nurses feeling dissatisfied with their work (Moghaddam et al. 2020; Souza et al. 2013a).

#### 3.5.3 To Do Good

Nurses found their work to be rewarding and gave them a sense of meaning (Flodén and Forsberg 2009; Simonsson et al. 2020). Feelings of satisfaction and joy occurred when the donation process had gone as expected, and the organs intended for donation had been successfully transplanted (Moghaddam et al. 2020; Starzomski et al. 2021). Organ donation was not a common occurrence, but when it did happen, many nurses welcomed it (Simonsson et al. 2020; Virgínio et al. 2014). The knowledge of being able to save other people and give them a new chance also contributed to joy and motivation among the nurses (Souza et al. 2013b). They felt proud of their work, as well as of their collaboration within the team (Simonsson et al. 2020).

#### 3.5.4 The Ability to Adapt

Nurses' goal was to work towards the best possible outcome for the patient, and in some cases, that meant organ donation (Victorino et al. 2019; Virgínio et al. 2014). Many nurses described experiencing a strange or unique feeling when a patient passed away, and the ongoing life‐sustaining treatment took on a new meaning (Simonsson et al. 2020). Shifting the focus from saving lives to preserving organs required a significant adjustment, and high demands were placed on the nurse's competence (Flodén, Berg, and Forsberg 2011; Gripewall et al. 2022; Simonsson et al. 2020). If the nurse was responsible for more patients at the same time, priorities could change (Moghaddam, Manzari, et al. 2018; Victorino et al. 2019). When the staff was insufficient, attention was directed towards patients with a better prognosis (Victorino et al. 2019). Some nurses found that the care became more complex, and their previous experiences and knowledge of the donation process could be crucial for how well they managed the transition from saving lives to preserving organs (Salehi et al. 2013).

#### 3.5.5 A Spiritual Aspect

Working with organ‐donation could create internal conflicts because the principle of the donation process went against their own spiritual and religious beliefs (Moghaddam et al. 2020; Starzomski et al. 2021; Virgínio et al. 2014). Some nurses believe that a person consists of both body and soul and that these should not be separated (Flodén and Forsberg 2009; Moghaddam et al. 2020; Virgínio et al. 2014). According to this, the patient is alive as long as their soul remains within the body, despite a confirmed diagnosis of brain death (Moghaddam et al. 2020).

## 4 Discussion

The results indicated that working with the donation process can be challenging on several levels, but there were also positive findings. The main findings of the results are discussed based on *The Process of Organ Donor Care* and its four phases (Figure 1), (Forsberg et al. 2014).

A main finding was the lack of knowledge among the nurses (Flodén and Forsberg 2009; Gripewall et al. 2022; Moghaddam et al. 2020; Salehi et al. 2013; Starzomski et al. 2021; Souza et al. 2013b), which had a negative impact on the nurses, and it was clear that there was a significant need for education (Moghaddam et al. 2020). Insufficient knowledge about brain death diagnosis led to stress and doubts among nurses, complicating their tasks in planning and leading nursing care (Gripewall et al. 2022; Moghaddam et al. 2020; Salehi et al. 2013). The lack of knowledge was a problem among physicians (Jawoniyi and Gormley 2015). A reason for this could be that the concept of death is interpreted differently depending on culture and religion (Jawoniyi and Gormley 2015; Randhawa 2012). Religious and spiritual beliefs could play a significant role in how the nurse perceives a brain‐dead patient (Flodén and Forsberg 2009; Moghaddam et al. 2020; Starzomski et al. 2021; Virgínio et al. 2014).

Nurses believed that the physiology behind the brain death diagnosis contradicted the patient's clinical appearance, making it emotionally challenging for them to accept that the patient was dead (Moghaddam, Pouresmaili, and Manzari 2018). According to Flodén, Persson, et al. (2011), it was revealed that nearly half of the included nurses only accepted brain death diagnosis when the clinical diagnosis was supplemented with a cerebral angiography. There was a significant ambiguity with brain death diagnosis, which is one reason why it was challenging for nurses to accept and confirm the brain death diagnosis (Flodén, Berg, and Forsberg 2011; Moghaddam, Pouresmaili, and Manzari 2018).

Teamwork is essential for optimising the care of an organ‐donor and meeting the needs of family members (Hunt and Murphy 2020). To ensure dignified and respectful care, the entire team needs to demonstrate decent and respectful behaviour, as advocated in phase three *‘Dignified and respectful care’* (Forsberg et al. 2014). Poor communication within the team and difficulties in collaboration could lead to issues such as indifference, lack of engagement, and dissatisfaction, which, in turn, could result in less effective work in the donation process (Araújo and Massarollo 2014).

The final phase, *‘Enabling a dignified farewell*,’ (Forsberg et al. 2014) concludes the donation process with the involvement of the personnel in a conference. Crisis processing was considered necessary (Flodén, Berg, and Forsberg 2011; Simonsson et al. 2020).

To respect and take care of family members was a central part in the donation process and constitutes phase two, *‘Respecting the Relatives*’ (Forsberg et al. 2014). It was evident in the results that the relationship with family members was a crucial but challenging aspect (Flodén, Berg, and Forsberg 2011; Gripewall et al. 2022; Simonsson et al. 2020; Starzomski et al. 2021). Ethical conflicts could arise when healthcare professionals initiated the conversation about organ donation with family members who were not fully aware of the seriousness of the situation (Moghaddam, Manzari, et al. 2018; Moghaddam et al. 2020). Therefore, it was essential to have clear communication and the ability to support family members when needed (Flodén, Berg, and Forsberg 2011; Gripewall et al. 2022). For family members, presence, support, care, and empathy from healthcare professionals were crucial (Kerstis and Widarsson 2020). It could, therefore, become problematic when nurses avoided contact with family members to cope with their own emotions and difficulties that could arise during the donation process (Souza et al. 2013b). Nurses' handling of emotions, such as avoiding contact with a specific patient or family member, was a common defence mechanism (Martins and Robazzi 2009).

When the focus shifted from saving lives to organ preservation treatment, nurses needed to use their adaptive capability, which could be challenging (Flodén, Berg, and Forsberg 2011; Gripewall et al. 2022; Simonsson et al. 2020; Virgínio et al. 2014). This can be applied to the first phase, *‘Safeguarding the dignity of the deceased person*,’ which describes the shift in focus and emphasises the importance of healthcare professionals making it clear to family members that the patient is deceased, and the focus should be on preserving the deceased person's dignity (Forsberg et al. 2014). Nurses describe that they work to make the transition extra clear for family members by communicating with the deceased person appropriately. By not talking *to* the deceased person, they signal to the family members that the patient is dead, and instead they talk *about* the deceased person (Orøy et al. 2015; Simonsson et al. 2020).

### 4.1 Strengths and Limitations

All included studies were conducted in an intensive care unit (ICU) in Sweden, Brazil, Norway, Iran, South Korea, and Canada. The ICU environment, healthcare system, and social structures could vary between the different countries. There are both cultural and religious differences which can affect transferability.

Recommendations for practice is to implement clinical guidelines for the organ donor process to ensure patient safety and improve nurses' working conditions. Due to the emotional stress during an organ donation process, there has to be a routine and time for debriefing after the donation process.

## 5 Conclusion

Nurses found the donation process challenging on multiple levels, highlighting the need for more knowledge and education. Effective teamwork and collegial support can ease the work, as well as clear guidelines and internal training. Ethical issues create stress and uncertainty. Addressing knowledge gaps can optimise the organisation of the donation process, enhancing nurses' competence, sense of meaning and job satisfaction.

## Author Contributions

N.T.R. and E.Å. contributed to the design of the study, collected the data, conducted the analysis, drafted the manuscript, and gave final approval of the version to be submitted.

## Funding

The authors have nothing to report.

## Conflicts of Interest

The authors declare no conflicts of interest.

## Data Availability

The data that support the findings of the study are available from the corresponding author upon reasonable request.
